# Supplementary material for: Effects of seated Tai Chi Yunshou on upper limb function among stroke patients in the subacute phase: A study protocol for a randomized controlled trial
Source: PLoS One. 2025 Nov 3;20(11):e0334823. doi: 10.1371/journal.pone.0334823 (PMC12582457; doi:10.1371/journal.pone.0334823)
Supplement: S2 Fig — (DOC) [file pone.0334823.s002.doc]

Figure. content for the schedule of enrolment, interventions, and assessments.*

|  | **Enrolment** | **Allocation** | **Intervention and follow up** | | |
| --- | --- | --- | --- | --- | --- |
| **TIMEPOINT**** | ***-2week*** | **-1week** | ***0*** | ***4week*** | ***8week*** |
| **ENROLMENT:** | ✓ |  |  |  |  |
| **Eligibility screen** | ✓ |  |  |  |  |
| **Informed consent** | ✓ |  |  |  |  |
| ***Demographics*** | ✓ |  |  |  |  |
| **Diagnosis** | ✓ |  |  |  |  |
| **Randomization and Allocation** |  | ✓ |  |  |  |
| **INTERVENTIONS:** |  |  |  |  |  |
| ***The experimental group*** |  |  |  |  |  |
| ***the control group*** |  |  |  |  |  |
| **ASSESSMENTS:** |  |  | ✓ | ✓ | ✓ |
| ***FMA-UE*** |  |  | ✓ | ✓ | ✓ |
| ***mTIS*** |  |  | ✓ | ✓ | ✓ |
| ***WMFT*** |  |  | ✓ | ✓ | ✓ |
| ***fNIRS*** |  |  | ✓ | ✓ | ✓ |
| ***BI*** |  |  | ✓ | ✓ | ✓ |
| ***Moca*** |  |  | ✓ | ✓ | ✓ |

Note: FMA-UE :Fugl-Meyer Upper Extremity Assessment; mTIS: Modified Trunk Impairment Scale; WMFT: Wolf Motor Function Test; fNIRS: Functional near-infrared spectroscopy; BI: Barthel Index. Moca: Montreal Cognitive Assessment.
