# Supplementary material for: Effects of seated Tai Chi Yunshou on upper limb function among stroke patients in the subacute phase: A study protocol for a randomized controlled trial
Source: PLoS One. 2025 Nov 3;20(11):e0334823. doi: 10.1371/journal.pone.0334823 (PMC12582457; doi:10.1371/journal.pone.0334823)
Supplement: S3 File — (DOC) [file pone.0334823.s003.doc]

**研究方案**

1 观察对象

1.1 病例来源

研究计划收集84例病例，均来源于2025年9月至2026年9月在上海中医药大学附属曙光医院康复医学科门诊或病房就诊的脑卒中患者。

1.2 纳入标准

1. 符合缺血性脑卒中或脑出血的诊断标准，经颅脑CT和（或）MRI检查证实；
2. 年龄在30-75岁之间；
3. MMSE评分＞24分；
4. 下肢Brunnstrom分期达到Ⅳ期或Ⅳ期以上；
5. 下肢肌力达4级或4级以上；
6. 能够独站并行走10米；
7. 家属及患者自愿配合治疗评估，签署知情同意书。

1.3 排除标准

1. 存在严重的糖尿病、高血压，心肝肾功能不全、恶性肿瘤；
2. 存在下肢静脉血栓、严重骨关节病变、下肢关节或肌肉损伤，处于骨折愈合期；
3. 存在视力障碍或其他可能导致平衡功能障碍的其他疾病；
4. 目前正在参与任何其他的临床研究。

1.4 剔除标准

1. 患者在试验期间违反试验方案；
2. 实验设备异常所致数据结果与实际具有偏差。

1.5观察病例的终止

1. 出现严重不良事件者，根据医生判断应该停止该病例临床观察者。
2. 病程中病情加重，或观察中出现了其他影响观察的病证，根据医生判断应该停止临床试验者。作无效病例处理。
3. 临床试验方案实施中发生了严重偏差，如依从性太差等。
4. 受试者在临床观察过程中不愿意继续进行临床观察，向主管医生或研究者提出退出临床观察的要求者。

1.6 病例的脱落与处理

1.6.1 脱落的标准

筛选合格进入随机化的患者，因故未完成本方案所规定的疗程及观察周期，作为脱落病例。

1.6.2 脱落病例的处理

1. 当患者脱落后，研究者应采取登门、预约随访、电话、信件等方式，尽可能与受试者联系，询问理由、记录最后一次训练时间并完成所能完成的评估项目。
2. 因出现不良反应、治疗无效等情况而退出观察病例，研究者应根据患者实际情况，采取相应的治疗措施。
3. 脱落病例均应妥善保存有关试验资料，既作留档，也是进行全分析集统计所需。脱落病人无需另补。

2 观察方案

2.1 分组

通过上海中医药大学附属曙光医院康复医学科门诊及病房筛选出符合纳入标准的脑卒中患者并签署知情同意书，确定入组病例84例并进行编号，随机分为2组，试验组42例、对照组42例.

2.2干预方案

试验组干预方案

试验组采用坐位云手+常规综合康复训练的方案。坐位云手由治疗师进行教学并指导或辅助患者完成训练；常规综合康复训练干预方案参考神经康复指南推荐的干预方法，包括拍打肌肉和挤压关节促进本体感觉、被动拉伸训练、主动和被动关节活动度训练、肌肉力量训练、体位转移训练及步行训练等。

每次训练30min，每日2次，每周训练5次，持续干预4周。

对照组干预方案

对照组仅采用常规综合康复训练的方案。

每次训练30min，每周训练5次，持续干预4周。

为保证受试者安全，采用治疗师与受试者一对一训练模式，受试者感觉疲劳或有任何不适即停止训练。

2.3 观察指标

详细记录所有受试者性别、年龄、身高、体重、婚姻状况、家庭住址、联系方式等个人信息并收集其临床特征，如：临床诊断、卒中类型、卒中位置和卒中发病时间等。

所有评估观察由专业的康复治疗师开展，分别在干预前、干预6周及干预后进行观察评估。

2.3.1 疗效评定指标

1. 主要疗效指标：

Fugl-Meyer Upper Extremity Assessment (FMA-UE)

1. 次要疗效指标：

Modified Trunk Impairment Scale (mTIS), Wolf Motor Function Test (WMFT), Functional near-infrared spectroscopy (fNIRS), Barthel Index(BI).

2.3.2 安全性指标

检查患者血压、心率、血氧饱和度并记录，试验期间出现的任何不良反应均应填写“不良事件表”,并追踪调查,详细记录处理经过及结果直到患者不适症状体征消失。追踪随访方式可以根据不良反应的轻重选择住院、门诊、家访、电话、通讯等多种形式。

2.4 统计方法

采用IBM SPSS 24.0软件进行统计学分析。描述性统计分析，定性指标以百分率描述，定量指标以均数、标准差描述。利用多重填补法对缺失数据进行填补。Shapiro-Wilk检验将用于检验基线和结果测量的连续变量的正态分布，连续变量将描述为正态分布的均值±SD或非正态分布的中位数;分类变量将被描述为频率。使用χ2检验或Fisher精确检验检验三组间分类变量的比较。采用重复测量的双因素方差分析检验组别与时间因素的主效应及组别与时间的交互效应。当时间-组交互作用显著时，进行一个简单的事后效应分析。所有检验的显著性水平均设定为0.05，并使用bonferroni校正方法进行多重比较校正。

2.5 质量控制方法

1. 实验前对所有参与本研究的受试患者进行健康宣教，详细交代研究操作过程，充分取得被试者的信任，争取患者合作，增强其依从性。所有病人均签署知情同意书。
2. 所有评估由3名治疗师完成，结果取平均值。

**Research Protocol**

Subjects of Observation

1.1 Source of Cases

The study plans to collect 84 cases, all of which will be sourced from stroke patients who visited the outpatient clinic or ward of the Rehabilitation Medicine Department at Shuguang Hospital Affiliated to Shanghai University of Traditional Chinese Medicine between September 2025 and September 2026.

1.2 Inclusion Criteria

(1) Diagnosis of ischemic stroke or cerebral hemorrhage confirmed by cranial CT and/or MRI;

(2) Age between 30 and 75 years;

(3) Mini-Mental State Examination (MMSE) score > 24;

(4) Brunnstrom stage IV or above for the lower limbs;

(5) Muscle strength of the lower limbs at grade 4 or above;

(6) Ability to stand independently and walk 10 meters;

(7) Voluntary participation in treatment evaluation by family members and patients, with signed informed consent.

1.3 Exclusion Criteria

(1) Presence of severe diabetes, hypertension, cardiac, hepatic, or renal insufficiency, or malignant tumors;

(2) Presence of lower extremity venous thrombosis, severe bone and joint lesions, lower extremity joint or muscle injuries, or in the fracture healing stage;

(3) Presence of visual impairments or other diseases that may lead to balance dysfunction;

(4) Currently participating in any other clinical studies.

1.4 Withdrawal Criteria

(1) Patients who violate the trial protocol during the trial;

(2) Data results deviating from reality due to abnormal experimental equipment.

1.5 Termination of Case Observation

(1) Patients experiencing serious adverse events, where clinical observation should be stopped according to the physician's judgment.

(2) Worsening of the disease during the course of illness or emergence of other conditions affecting observation, where clinical trials should be stopped according to the physician's judgment. Such cases are considered invalid.

(3) Significant deviations occurring during the implementation of the clinical trial protocol, such as poor compliance.

(4) Participants unwilling to continue clinical observation during the process and requesting withdrawal from the clinical observation to the attending physician or researcher.

1.6 Dropout and Management

1.6.1 Criteria for Dropout

Patients who are screened and randomized but fail to complete the prescribed treatment course and observation period for reasons specified in the protocol are considered dropouts.

1.6.2 Management of Dropout Cases

(1) When a patient drops out, researchers should attempt to contact the participant through home visits, scheduled follow-ups, phone calls, letters, etc., to inquire about the reasons, record the last training session, and complete any assessable items.

(2) For patients who withdraw due to adverse reactions or ineffective treatment, researchers should take appropriate therapeutic measures based on the patient's actual condition.

(3) Relevant trial data for dropout cases should be properly stored for archiving and for the full analysis set statistics. No replacement is needed for dropout patients.

Observation Plan

2.1 Grouping

Stroke patients meeting the inclusion criteria are screened from the outpatient clinic and ward of the Rehabilitation Medicine Department at Shuguang Hospital Affiliated to Shanghai University of Traditional Chinese Medicine, and informed consent is obtained. A total of 84 eligible cases are identified, numbered, and randomly divided into two groups: 42 in the experimental group and 42 in the control group.

2.2 Intervention Plan

Intervention Plan for the Experimental Group

The experimental group receives a combination of seated Cloud Hands exercise and conventional comprehensive rehabilitation training. The seated Cloud Hands exercise is taught and guided or assisted by a therapist. The conventional comprehensive rehabilitation training intervention follows methods recommended in neurorehabilitation guidelines, including muscle tapping and joint compression to promote proprioception, passive stretching exercises, active and passive joint range of motion exercises, muscle strength training, positional transfer training, and walking training.

Each training session lasts 30 minutes, twice daily, five times a week, for a continuous intervention period of 4 weeks.

Intervention Plan for the Control Group

The control group receives only conventional comprehensive rehabilitation training.

Each training session lasts 30 minutes, five times a week, for a continuous intervention period of 4 weeks.

To ensure participant safety, a one-on-one training model is adopted between the therapist and the participant. Training is stopped immediately if the participant feels fatigued or experiences any discomfort.

2.3 Observation Indicators

Detailed records are kept of all participants' personal information, including gender, age, height, weight, marital status, home address, contact information, etc., and their clinical characteristics are collected, such as clinical diagnosis, type of stroke, location of stroke, and time of stroke onset.

All assessments and observations are conducted by professional rehabilitation therapists before the intervention, at 6 weeks into the intervention, and after the intervention.

2.3.1 Efficacy Evaluation Indicators

(1) Primary Efficacy Indicator:

Fugl-Meyer Upper Extremity Assessment (FMA-UE)

(2) Secondary Efficacy Indicators:

Modified Trunk Impairment Scale (mTIS), Wolf Motor Function Test (WMFT), Functional near-infrared spectroscopy (fNIRS), Barthel Index (BI).

2.3.2 Safety Indicators

Patients' blood pressure, heart rate, and blood oxygen saturation are checked and recorded. Any adverse events occurring during the trial are documented in an "Adverse Event Form" and followed up. Detailed records of the handling process and outcomes are maintained until the patient's discomforting symptoms and signs disappear. Follow-up methods can include hospitalization, outpatient visits, home visits, phone calls, communication, etc., depending on the severity of the adverse reaction.

2.4 Statistical Methods

Statistical analysis is performed using IBM SPSS 24.0 software. Descriptive statistical analysis is conducted, with qualitative indicators described as percentages and quantitative indicators as means and standard deviations. Missing data are imputed using the multiple imputation method. The Shapiro-Wilk test is used to test the normal distribution of continuous variables at baseline and outcome measurements. Continuous variables are described as mean ± SD for normal distributions or median for non-normal distributions; categorical variables are described as frequencies. The χ2 test or Fisher's exact test is used to compare categorical variables among the three groups. A repeated-measures two-way ANOVA is used to test the main effects of group and time factors and their interaction. When the time-group interaction is significant, a simple post-hoc effect analysis is performed. The significance level for all tests is set at 0.05, and the Bonferroni correction method is used for multiple comparison corrections.

2.5 Quality Control Methods

(1) All patients participating in this study receive health education before the experiment, with detailed explanations of the study procedures to gain their trust, cooperate with patients, and enhance their compliance. All patients sign informed consent forms.

(2) All assessments are completed by three therapists, with results averaged.
