# Supplementary material for: Effects of seated Tai Chi Yunshou on upper limb function among stroke patients in the subacute phase: A study protocol for a randomized controlled trial
Source: PLoS One. 2025 Nov 3;20(11):e0334823. doi: 10.1371/journal.pone.0334823 (PMC12582457; doi:10.1371/journal.pone.0334823)
Supplement: S4 File — (DOC) [file pone.0334823.s004.doc]

**Research Protocol**

Subjects of Observation

1.1 Source of Cases

The study plans to collect 84 cases, all of which will be sourced from stroke patients who visited the outpatient clinic or ward of the Rehabilitation Medicine Department at Shuguang Hospital Affiliated to Shanghai University of Traditional Chinese Medicine between September 2025 and September 2026.

1.2 Inclusion Criteria

(1) Diagnosis of ischemic stroke or cerebral hemorrhage confirmed by cranial CT and/or MRI;

(2) Age between 30 and 75 years;

(3) Mini-Mental State Examination (MMSE) score > 24;

(4) Brunnstrom stage IV or above for the lower limbs;

(5) Muscle strength of the lower limbs at grade 4 or above;

(6) Ability to stand independently and walk 10 meters;

(7) Voluntary participation in treatment evaluation by family members and patients, with signed informed consent.

1.3 Exclusion Criteria

(1) Presence of severe diabetes, hypertension, cardiac, hepatic, or renal insufficiency, or malignant tumors;

(2) Presence of lower extremity venous thrombosis, severe bone and joint lesions, lower extremity joint or muscle injuries, or in the fracture healing stage;

(3) Presence of visual impairments or other diseases that may lead to balance dysfunction;

(4) Currently participating in any other clinical studies.

1.4 Withdrawal Criteria

(1) Patients who violate the trial protocol during the trial;

(2) Data results deviating from reality due to abnormal experimental equipment.

1.5 Termination of Case Observation

(1) Patients experiencing serious adverse events, where clinical observation should be stopped according to the physician's judgment.

(2) Worsening of the disease during the course of illness or emergence of other conditions affecting observation, where clinical trials should be stopped according to the physician's judgment. Such cases are considered invalid.

(3) Significant deviations occurring during the implementation of the clinical trial protocol, such as poor compliance.

(4) Participants unwilling to continue clinical observation during the process and requesting withdrawal from the clinical observation to the attending physician or researcher.

1.6 Dropout and Management

1.6.1 Criteria for Dropout

Patients who are screened and randomized but fail to complete the prescribed treatment course and observation period for reasons specified in the protocol are considered dropouts.

1.6.2 Management of Dropout Cases

(1) When a patient drops out, researchers should attempt to contact the participant through home visits, scheduled follow-ups, phone calls, letters, etc., to inquire about the reasons, record the last training session, and complete any assessable items.

(2) For patients who withdraw due to adverse reactions or ineffective treatment, researchers should take appropriate therapeutic measures based on the patient's actual condition.

(3) Relevant trial data for dropout cases should be properly stored for archiving and for the full analysis set statistics. No replacement is needed for dropout patients.

Observation Plan

2.1 Grouping

Stroke patients meeting the inclusion criteria are screened from the outpatient clinic and ward of the Rehabilitation Medicine Department at Shuguang Hospital Affiliated to Shanghai University of Traditional Chinese Medicine, and informed consent is obtained. A total of 84 eligible cases are identified, numbered, and randomly divided into two groups: 42 in the experimental group and 42 in the control group.

2.2 Intervention Plan

Intervention Plan for the Experimental Group

The experimental group receives a combination of seated Cloud Hands exercise and conventional comprehensive rehabilitation training. The seated Cloud Hands exercise is taught and guided or assisted by a therapist. The conventional comprehensive rehabilitation training intervention follows methods recommended in neurorehabilitation guidelines, including muscle tapping and joint compression to promote proprioception, passive stretching exercises, active and passive joint range of motion exercises, muscle strength training, positional transfer training, and walking training.

Each training session lasts 30 minutes, twice daily, five times a week, for a continuous intervention period of 4 weeks.

Intervention Plan for the Control Group

The control group receives only conventional comprehensive rehabilitation training.

Each training session lasts 30 minutes, five times a week, for a continuous intervention period of 4 weeks.

To ensure participant safety, a one-on-one training model is adopted between the therapist and the participant. Training is stopped immediately if the participant feels fatigued or experiences any discomfort.

2.3 Observation Indicators

Detailed records are kept of all participants' personal information, including gender, age, height, weight, marital status, home address, contact information, etc., and their clinical characteristics are collected, such as clinical diagnosis, type of stroke, location of stroke, and time of stroke onset.

All assessments and observations are conducted by professional rehabilitation therapists before the intervention, at 6 weeks into the intervention, and after the intervention.

2.3.1 Efficacy Evaluation Indicators

(1) Primary Efficacy Indicator:

Fugl-Meyer Upper Extremity Assessment (FMA-UE)

(2) Secondary Efficacy Indicators:

Modified Trunk Impairment Scale (mTIS), Wolf Motor Function Test (WMFT), Functional near-infrared spectroscopy (fNIRS), Barthel Index (BI).

2.3.2 Safety Indicators

Patients' blood pressure, heart rate, and blood oxygen saturation are checked and recorded. Any adverse events occurring during the trial are documented in an "Adverse Event Form" and followed up. Detailed records of the handling process and outcomes are maintained until the patient's discomforting symptoms and signs disappear. Follow-up methods can include hospitalization, outpatient visits, home visits, phone calls, communication, etc., depending on the severity of the adverse reaction.

2.4 Statistical Methods

Statistical analysis is performed using IBM SPSS 24.0 software. Descriptive statistical analysis is conducted, with qualitative indicators described as percentages and quantitative indicators as means and standard deviations. Missing data are imputed using the multiple imputation method. The Shapiro-Wilk test is used to test the normal distribution of continuous variables at baseline and outcome measurements. Continuous variables are described as mean ± SD for normal distributions or median for non-normal distributions; categorical variables are described as frequencies. The χ2 test or Fisher's exact test is used to compare categorical variables among the three groups. A repeated-measures two-way ANOVA is used to test the main effects of group and time factors and their interaction. When the time-group interaction is significant, a simple post-hoc effect analysis is performed. The significance level for all tests is set at 0.05, and the Bonferroni correction method is used for multiple comparison corrections.

2.5 Quality Control Methods

(1) All patients participating in this study receive health education before the experiment, with detailed explanations of the study procedures to gain their trust, cooperate with patients, and enhance their compliance. All patients sign informed consent forms.

(2) All assessments are completed by three therapists, with results averaged.
