# Supplementary figures and images for: Effects of seated Tai Chi Yunshou on upper limb function among stroke patients in the subacute phase: A study protocol for a randomized controlled trial
Source: PLoS One. 2025 Nov 3;20(11):e0334823. doi: 10.1371/journal.pone.0334823 (PMC12582457; doi:10.1371/journal.pone.0334823)

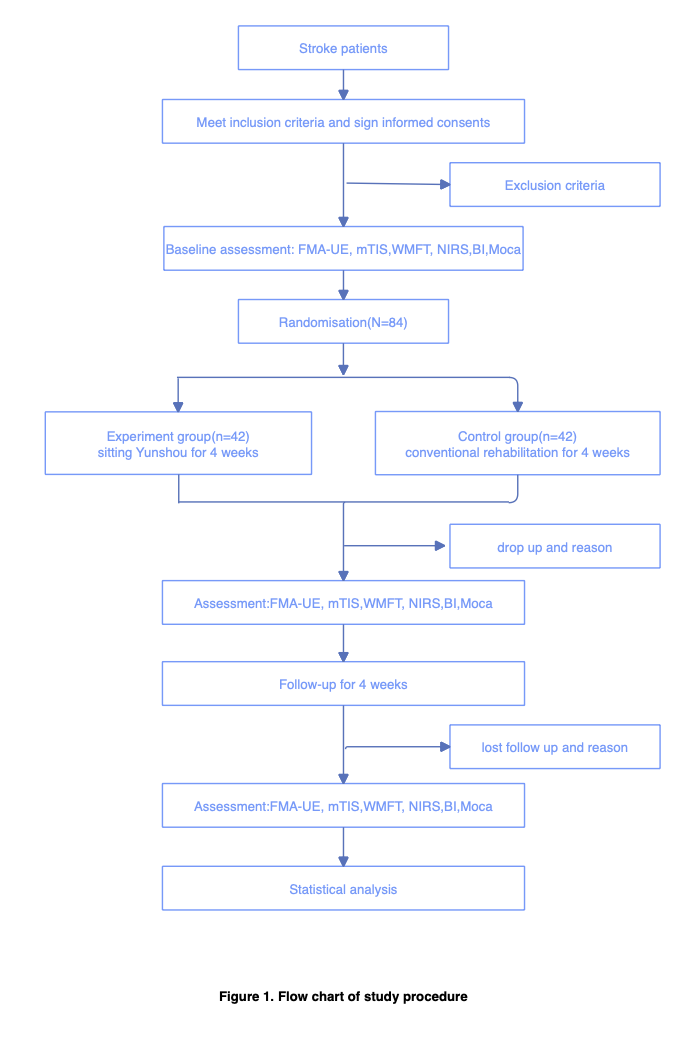

Supplement: S5 Fig — (PNG) [file pone.0334823.s005.png]
