# Supplementary material for: Effects of seated Tai Chi Yunshou on upper limb function among stroke patients in the subacute phase: A study protocol for a randomized controlled trial
Source: PLoS One. 2025 Nov 3;20(11):e0334823. doi: 10.1371/journal.pone.0334823 (PMC12582457; doi:10.1371/journal.pone.0334823)
Supplement: Attachment — Submitted filename: Response to reviewers_seated Yunshou1.docx [file pone.0334823.s007.docx]

Dear Editor and Reviewers

We sincerely appreciate the reviewers’ thoughtful comments and valuable suggestions on our manuscript. We are also grateful for the opportunity to revise our paper. All points raised by the Editors and reviewers have been addressed in the point-by-point response below and incorporated into the revised manuscript using track changes with red highlighting. In addition, we have restructured the article and supplemented the content as necessary to ensure full compliance with the CONSORT guidelines. We believe that our revisions have substantially improved the manuscript and hope that it now meets the journal’s standards for publication.

Yours faithfully,

Jiming Tao

**Editor**

**Response**
Q: (1) Please ensure that your manuscript meets PLOS ONE's style requirements, including those for file naming.

A: Thank you very much for your suggestion. We have changed the format according to the journal requirements.

Q:(2) Thank you for stating the following financial disclosure:“Key Supported Discipline in Rehabilitation Medicine within the Shanghai Health and Health System（2023ZDFC0301）”. Please state what role the funders took in the study. If the funders had no role, please state: "The funders had no role in study design, data collection and analysis, decision to publish, or preparation of the manuscript."

A:Thank you very much for your suggestion. We have stated "The funders had no role in study design, data collection and analysis, decision to publish, or preparation of the manuscript."(See Line 427-430).

Q:(3) Thank you for stating the following in your Competing Interests section:

“No Conflict of Interest”. Please complete your Competing Interests on the online submission form to state any Competing Interests. If you have no competing interests, please state "The authors have declared that no competing interests exist."

A: We have stated "The authors have declared that no competing interests exist." (See Line 425-426).

Q:(4) When completing the data availability statement of the submission form, you indicated that you will make your data available on acceptance. We strongly recommend all authors decide on a data sharing plan before acceptance, as the process can be lengthy and hold up publication timelines. Please note that, though access restrictions are acceptable now, your entire data will need to be made freely accessible if your manuscript is accepted for publication. This policy applies to all data except where public deposition would breach compliance with the protocol approved by your research ethics board. If you are unable to adhere to our open data policy, please kindly revise your statement to explain your reasoning and we will seek the editor's input on an exemption. Please be assured that, once you have provided your new statement, the assessment of your exemption will not hold up the peer review process.

A: Thank you very much for your suggestion. We confirm our full adherence to the journal’s open data policy. All data required to reproduce the findings presented in this study will be made freely and publicly accessible upon acceptance of the manuscript. We have selected [Figshare] for data deposition. The shared materials will include [de-identified raw data, analysis scripts, and detailed protocols].The data will be published under a CC-BY 4.0 license and assigned a persistent DOI. We will promptly provide the DOI and a direct access link to the editorial office as soon as the deposition is complete, to avoid any delay in the publication process.

Q:(5) Please include captions for your Supporting Information files at the end of your manuscript, and update any in-text citations to match accordingly. Please see our Supporting Information guidelines for more information: <http://journals.plos.org/plosone/s/supporting-information>.

A: We thank the reviewer for this helpful reminder. We have now included detailed captions for all Supporting Information files at the end of the manuscript and have updated all relevant in-text citations to correspond accurately to these files. The changes have been made in accordance with the journal’s Supporting Information guidelines. (See Line 431-440).

Q:(6) If the reviewer comments include a recommendation to cite specific previously published works, please review and evaluate these publications to determine whether they are relevant and should be cited. There is no requirement to cite these works unless the editor has indicated otherwise.

A: Thank you very much for your reminder. We have cited the article according to the reviewer’s suggestion. (See Line 162 and Line 488-489).

**Reviewer 1**

Q:(1) The sample size design is written reasonably clearly with anticipated dropouts.

The effect size appears to have been calculated with a standard deviation of 2 given the current data in the sample size section. Is that the only source of past data? What about the literature, if any?

A: We thank the reviewer for their attentive comment regarding the sample size calculation. The effect size was indeed estimated based on the standard deviation observed in our pilot data, as presented in Reference 18. This reference provides preliminary results from a comparable population using the same assessment methods, ensuring clinical and methodological consistency with our current study(See Line 162 and Line 488-489).

Q:(2) The initial patient assessment appears to be ongoing as per the sections on Participants and Exclusion criteria which appear to be written in the past tense. There should be an updated writing as to the exact assessments being made for these initial assessments.

A: We thank the reviewer for this careful observation. The reviewer is correct to note the inconsistent use of tense. We have now revised the Participants and Exclusion Criteria sections to be written consistently in the future tense, to accurately reflect that initial patient assessments will be conducted at the time of enrolment (See Line 111-140).

Q:(3) [Randomization and blinding are reasonable. Primary and secondary endpoints are presented. The statistical analysis is expected to be a repeated measures ANOVA with alternative non parametric Wilcoxon rank sum. However, a repeated measures nonparametric alternative should be in place as well, should it be needed. The time line figure looks reasonable.](http://journals.plos.org/plosone/s/supporting-information.)

A: Thank you very much for your reminder. We have increased a repeated measures nonparametric alternative if needed (See Line 331-338).

Q: (4) With such a small sample is imputation as noted in the protocol really reasonable? If so, presumably there will be an imputed and complete data set analysis for quality control purposes and data interpretation checks.

A： We thank the reviewer for raising this important methodological point regarding the use of imputation for a small sample size. We agree that this requires careful consideration and transparent reporting. We have corrected the statistic method according the reviewer’s suggestion (See Line 358-362)

Q: (5) There should be a section on data management and quality control. Section 2.5 in the protocol supplement requires more detail on the actual quality control of the data and data flow.

A: Thank you very much for your reminder. We have added the data management and quality control(See Line 310-325 and Line 344-357).

Q: (6) Planned management of dropout cases is well planned as per the supplemental protocol.

A：We sincerely thank the reviewer for their positive feedback on our planned management of dropout cases. We are pleased that our approach is considered well planned(See Line 339-343).

**Reviewer 2**

Q: (1) The theoretical framework remains descriptively broad and lacks integration with established neurorehabilitation models, such as Dynamic Systems Theory, Task-Oriented Training, or the International Classification of Functioning (ICF). The authors suggest Tai Chi optimizes interhemispheric connectivity, yet do not clearly define mechanisms through which seated Tai Chi Yunshou would differ in this effect from other bilateral or trunk-focused interventions (e.g., bilateral arm training or PNF). The use of “mind-body coordination” and “cognitive-motor synergy” is conceptually promising but needs more rigorous operationalization.

A: Thank you very much for your reminder. We have added the Dynamic Systems Theory, Task-Oriented Training, or the International Classification of Functioning (ICF) and try discuss the difference from bilateral interventions(See Line 77 to 91 and Line 364-386).

Q: (2) While randomization is computer-generated and opaque envelopes are used, the lack of block randomization or stratification (e.g., based on stroke severity or dominant hand) may compromise balance across groups.

A: Thank you very much for your reminder. We have added block randomization(See Line 172-175)

Q: (3) The control group receives conventional therapy, including a broad range of interventions (acupuncture, Tuina, speech therapy), which may vary widely across patients. Without standardized delivery, this introduces treatment heterogeneity, weakening internal validity. The authors do not quantify therapy dose equivalently across groups (i.e., whether total contact time is matched), risking confounding from unequal therapeutic exposure.

A: Thank you very much for your reminder. We have corrected the therapy part to ensure the standardized delivery and quantify therapy dose equivalently across groups(See Line 200-212).

Q: (4) The inclusion of fNIRS is forward-looking, but the analysis plan lacks detail. No hypotheses are presented about expected regional activation patterns (e.g., M1, SMA, DLPFC), and no preprocessing or statistical correction strategy (e.g., FDR, Bonferroni) is outlined for handling fNIRS’ multiple comparisons problem.

A：Thank you very much for your reminder. We have added hypotheses and preprocession (See Line 281-292).

Q: (5) The manuscript refers repeatedly to “enhanced interhemispheric connectivity” and “functional remapping,” yet no objective functional connectivity analyses (e.g., correlation or coherence matrices from fNIRS) are proposed.

A：Thank you very much for your reminder. We have added correlation from fNIRS(See Line 281-292).

Q: (6) The authors claim this is a mind-body therapy but do not measure cognitive, attentional, or emotional outcomes (e.g., MoCA, Stroop test, or depression/anxiety scales), leaving the “mind” component entirely theoretical.

A：Thank you very much for your reminder. We added MoCA as a secondary measurement to assess the cognitive outcome(See Line 300-309).
